# Serum Derived Extracellular Vesicles Mediated Delivery of Synthetic miRNAs in Human Endothelial Cells

**DOI:** 10.3389/fmolb.2021.636587

**Published:** 2021-03-26

**Authors:** Marta Tapparo, Margherita Alba Carlotta Pomatto, Maria Chiara Deregibus, Elli Papadimitriou, Claudia Cavallari, Sergio D’Antico, Federica Collino, Giovanni Camussi

**Affiliations:** ^1^ Department of Medical Sciences, Molecular Biotechnology Center, University of Torino, Turin, Italy; ^2^ 2i3T SCARL, University of Torino, Turin, Italy; ^3^ Department of Molecular Biotechnology and Health Sciences, University of Torino, Turin, Italy; ^4^ Blood Bank, A.O.U. Città della Salute e della Scienza, Turin, Italy; ^5^ Laboratory of Translational Research in Paediatric Nephro-Urology, Fondazione Ca’ Granda IRCCS Ospedale Maggiore Policlinico, of Milano, Milan, Italy

**Keywords:** extracellular vesicles, miRNA loading, angiogenesis, biological fluid, surface proteins, binding activity

## Abstract

Extracellular vesicles (EVs) have emerged in the last decades as a cell-to-cell communication mechanism. One of their mechanism of action is the direct delivery of their cargo, composed of bioactive molecules to target cells. Different methods (direct electroporation, cell transfection, chemical transfection) were developed to vehicle therapeutic molecules through EVs. However, most of these techniques presented some limitations such as EV disruption and aggregation. In the present study, we demonstrated that a direct temperature-controlled co-incubation of EVs with defined miRNAs is a stable method to deliver information to target cells without affecting EV constitutive content. We chose serum as an easy and abundant source of EVs applicable to autologous treatment after EV modification. Exogenous cel-miR-39 loaded on serum EVs (SEVs) was taken up by human endothelial cells, demonstrating an adequate miRNA loading efficacy based on the co-incubation method. Moreover, SEVs co-incubation with the angiomiRNA-126 (miR-126) enhanced their angiogenic properties *in vitro* and *in vivo* by increasing the capacity to induce capillary-like structure formation of human endothelial cells. MiR-126 loaded EVs were also shown to stimulate mouse endothelial cells to invade Matrigel plugs and create more vessels with respect to the EV naive counterpart. When SEVs were loaded with miR-19b, an anti-angiogenic miRNA, they were able to reduce Vascular endothelial growth factors (VEGF) pro-angiogenic capacity, supporting the selective biological effect mediated by the carried miRNA. Lastly, we identified Annexin A2 (ANXA2) as one of the molecules involved in the exogenous RNA binding to serum EV surface, favoring miRNA delivery to target endothelial cells for potential therapeutic application.

## Introduction

In the last decades, the discovery of extracellular vesicles (EVs) as a cell-to-cell communication mechanism opened to new applications in regenerative medicine and in cancer therapy. The use of EVs instead of cells, is a promising strategy because EVs retain most of the properties of their originating cells, but do not show side effects related to immune response (Mentkowski et al., 2018). Moreover, EVs ensure a stable environment for the delivery of selected molecules such as miRNAs and proteins, that are incorporated into EVs by a complex and not yet completely recognized machinery (Colombo et al., 2014; Iavello et al., 2016).

The serum has been described as an easy source of EVs, containing both RNAs and proteins (Caradec et al., 2014; Cheng et al., 2014) currently used as prognostic biomarkers in cancers (Xu et al., 2018) and chronic diseases patients (Grassmann et al., 2014; Zhang et al., 2014). In a recent study, Cavallari and colleagues (Cavallari et al., 2017) demonstrated the pro-angiogenic capacity of EVs collected from the sera of healthy subjects. In this study the authors showed that serum EVs (SEVs) were effective in inducing the formation of capillary-like structure *in vitro* and improving neo-angiogenesis in a model of acute hind limb ischemia (Cavallari et al., 2017).

The loading of exogenous molecules into EVs, with known biological effect, is an attractive new tool that can be applied in different therapeutic fields. Different molecules, such as small RNAs, proteins, and antitumor drugs were recently investigated as supplementary cargo conveyed by EVs (Zhuang et al., 2011; El-Andaloussi et al., 2012; Yim et al., 2016; Kanchanapally et al., 2019). Several loading methods with specific therapeutic molecules, in particular microRNAs (miRNAs) and small interfering RNAs (siRNAs), have been developed in the last decade (Yang and Li, 2017; Usman et al., 2018). Studies were conducted with genetically modified cell derived-EVs (Akao et al., 2011; Wang et al., 2016; Tapparo et al., 2019) or with EVs directly engineered by electroporation or chemical transfection (Shtam et al., 2013; Haney et al., 2015). Electroporation of EVs was shown to be effective in the enrichment of selected miRNAs but can induce mimic aggregation (Kooijmans et al., 2013) and possible damage of the membrane and surface molecules of EVs (Lamichhane et al., 2015; Johnsen et al., 2016). Co-incubation is a good alternative in order to modify the biological activity of EVs by enrichment of defined molecules without affecting their constitutive content. Passive loading with lipophilic drug or cholesterol conjugated siRNAs (Fuhrmann et al., 2015; O’Loughlin et al., 2017; Didiot et al., 2018) showed the capability of EVs to deliver exogenous material to specific target cells. O’Loughlin and colleagues demonstrated that incubation of siRNA facilitated a concentration-dependent silencing of human antigen R (HuR), a therapeutic target in cancer, in EV-treated cells (O’Loughlin et al., 2017). Moreover, in another study, a hydrophobically modified siRNA co-incubated with glioblastoma derived exosomes, was delivered to the brain, inducing the downregulation of the *Huntingtin* gene (Didiot et al., 2018). Unfortunately, most of the findings did not provide evidence on the efficacy of the loaded EVs.

Studies on the regulation of cargo loading into EVs became very important to provide the basis for using EVs as a vehicle to transport and deliver therapeutic molecules (Villarroya-Beltri et al., 2014; Janas et al., 2015). Endogenous miRNA loading could be dependent on miRNA specific sequence motif or sequence modification (Villarroya-Beltri et al., 2013; Squadrito et al., 2014; Wani and Kaul, 2018). On the other hand, the binding can be also miRNA sequence-independent, being mediated by the capability of some RNA-binding proteins to recognize the secondary structure of a miRNA (van Kouwenhove et al., 2011). In this context, the presence of specific proteins on EV surface can facilitate miRNA packaging into EVs (Turchinovich et al., 2011; Hagiwara et al., 2015; Iavello et al., 2016).

The aim of the present study was to investigate the efficacy of a simple co-incubation protocol to modify SEVs biological activity with miRNA mimics. Loading efficacy and transfer experiments were validated using the exogenous cel-miR-39 mimic from *C. Elegans*. We investigate the different effects of the angiomiR-126 (miR-126) (Tiwari et al., 2018) and the angiogenesis inhibitor, miR-19b (Liang et al., 2018) co-incubated with SEVs both *in vitro* and *in vivo*. Moreover, the active role of proteins on EV surface on exogenous RNA binding was evaluated.

## Material and Methods

### SEV Isolation and Characterization

Human SEVs were isolated from sera of healthy donors (n = 10) provided by the Blood Bank of “Città della Salute e della Scienza di Torino” (Cavallari et al., 2017). All donors gave informed consent, and the study was approved by the local Ethical Review Board. Samples were centrifuged at 3,000 g for 30 min to remove cell debris followed by microfiltration with Millipore 0.22 μm filters and then subjected to the first ultracentrifugation at 100,000 g (Beckman Coulter Optima L-90K ultracentrifuge, Brea, CA, United States) for 3 h at 4°C. To wash SEVs, the second ultracentrifugation with Phosphate Buffer Saline (PBS) was performed at 100,000 g for 3 h. Collected SEVs were resuspended in serum-free Roswell Park Memorial Institute (RPMI) medium (Lonza, Basel, Switzerland) supplemented with 1% Dimethyl sulfoxide (DMSO) (Sigma-Aldrich, St. Louis, MO, United States) and stored at −80°C.

SEV number and size distribution were quantified by Nanosight LM300 instrument (NanoSight Ltd., Amesbury, United Kingdom) equipped with NTA 3.2 Analytical Software.

The cytofluorimetric analysis was performed by Guava easyCyte Flow Cytometer (Millipore, Billerica, MA, United States) and analyzed with InCyte software using the following fluorescein isothiocyanate (FITC)-, phycoerythrin (PE)- or–allophycocyanin (APC) conjugated antibodies: CD44, CD105, α4 integrin, α5 integrin, α6 integrin, CD16, CD3, CD4, CD31 (Miltenyi Biotec, Bergisch Gladbach, Germany), CD73, CD29, CD90, CD34, HLA-DR, CD14, CD20, KDR, CD41, P-Selectin, CD42B (BD Biosciences). FITC, PE or APC mouse isotypic IgG (Miltenyi Biotec) were used as controls. Briefly, SEVs (5 × 10^8^ particles) were resuspended in 100 µl of 0.1 µm filtered PBS 1X and incubated with the selected antibodies for 15 min at 4°C; then sample were diluted in 200 µl filtered PBS 1X and were acquired.

### Transmission Electron Microscopy and *in situ* Hybridization

SEVs were analyzed by electron microscopy as previously described (Pomatto et al., 2019). After 20 min of adhesion on 200 mesh nickel formvar carbon-coated grids (Electron Microscopy Science, Hatfield, Pennsylvania, United States), samples were fixed with 2.5% glutaraldehyde containing 2% sucrose for 15 min and extensively washed with distilled water. SEVs were negatively stained with Nano-W and NanoVan (Nanoprobes, Yaphank, New York, United States).

In selected experiments *in situ* hybridization was performed to show the binding of cel-miR-39 to SEVs, using a miRCURY LNA detection probe biotinylated at 5′ of its sequence (Exiqon, Vedbaek, Denmark) following manufacturer’s instruction. Briefly, EVs were prefixed with 4% paraformaldehyde for 15 min at room temperature on a formvar-carbon coated grid. The hybridization with the biotinylated LNA probe for cel-miR-39 (Exiqon, Vedbaek, Denmark) was carried out at 20–22°C below the melting temperature of the probe overnight (ON) after incubation in a pre-hybridization buffer for 2 h at the temperature of annealing of the probe (59°C) in a water bath (Collino et al., 2010). The day after the samples were incubated with 10 nm gold-conjugated avidin (Nanoprobes) and were negatively stained with Nano-W and NanoVan (Nanoprobes) (Collino et al., 2010). Samples were analyzed by a Jeol JEM 1010 electron microscope (Jeol, Tokyo, Japan).

### SEV Loading With Synthetic miRNAs

SEVs (100 × 10^8^ particles/100 µl) were co-incubated for 1 h at 37°C in RPMI medium (Lonza) with synthetic mimic cel-miR-39 (SEV + cel-miR-39) or hsa-miR-126–3p (miR-126, 100 pmol) (SEV + miR-126) or hsa-miR-19b-3p (miR-19b, 100 pmol) (SEV + miR-19b) according to the different experiments (Qiagen, Venlo, Netherlands). SEVs and mimic alone were included as a control in each experiment. After incubation, SEVs, mimic, and SEV + mimic samples were washed twice with Amicon® Ultra 15 ml 50 kDa filter (Merck-Millipore, Darmstadt, Germany) by centrifugation at 3,000 g for 15 min at RT. For *in vivo* experiments, samples were cleaned by dialysis using a cellulose membrane (Float-a-Lyzer G2-Sigma) with a 100 kDa cut-off in a physiologic solution. Dialysis wash was performed for 4 h and the solution was replaced and left for further 16 h at 4°C, to allow a correct exchange between the external and internal environments of the membrane.

### Incorporation and Transfer Assay of Modified SEVs Into Human Endothelial Cells

To determine SEV incorporation, particles were labeled with 250 nM Vybrant Dil dye (Molecular probe-Invitrogen, Carlsbad, CA, United States) and co-incubated with FITC conjugated - AllStars Negative Control siRNA (siRNA-FITC) (Qiagen) according to the SEV/mimic ratio described before. SEVs and siRNA-FITC alone were used as control. Human Umbilical Vein Endothelial Cells (HUVEC) were treated 24 h in the Endothelial Basal Medium (EBM) (Lonza) with a labeled SEVs co-incubated with fluorescent mimic. SEV incorporation was confirmed by confocal microscopy (Zeiss LSM 5 Pascal Model Confocal Microscope-Carl Zeiss International, Oberkochen, Germany).

To evaluate the transfer of mimics to target cells, HUVEC were treated in EBM (Lonza) with SEVs, mimic alone or miRNA loaded SEVs (SEV + miR). In cel-miR-39 transfer experiments, HUVEC were treated for 16–18 h (ON), and then the medium was changed, and cells left to grow for 5 or 24 h. In experiments with hsa-mir-126-3p, HUVEC were incubated 5 h or ON, mimicking the timing of *in vitro* experiments. At the selected time points, endothelial cells were lyzed in Trizol (Ambion, Thermo Fisher Scientific, Waltham, MA, United Kingdom) and RNA isolation was performed following the manufacturer’s protocol. miRNA expression was analyzed by RNA retrotranscription and Real-time PCR using miScript PCR System (Qiagen) according to the manufacturer’s protocol. Specific primers for cel-miR-39 (F: CAC​CGG​GTG​TAA​ATC​AGC​TTG) and has-miR-126-3p (F: TCG​TAC​CGT​GAG​TAA​TAA​TGC​G) were used. RNU48 (F: AAC​TCT​GAG​TGT​GTC​GCT​GAT​G) was used as reference. miRNA expression was expressed as Relative quantification (RQ) ±SD, calculated according the 2^−ΔΔCt^ formula.

### Trypsin Treatment of siRNA-FITC Loaded SEVs

SEVs were co-incubated as described above with siRNA-FITC and treated 1 h a 37°C with a serial dilution of trypsin (from 1:1,000 to 1:2,500 of 1X Trypsin solution; 170 U/ml, Sigma). The fluorescent signal was acquired with the GloMax Multi Detection System plate reader (Promega, Fitchburg, WI, United States) and data were expressed as Fluorescence Intensity ± SD. Experiments were performed 3 times in duplicates.

### Immunoprecipitation and Western Blot on SEVs

Protein immunoprecipitation was conducted as follows. SEVs, SEVs co-incubated with cel-miR-39 mimic and single mimic were lyzed in radioimmunoprecipitation assay (RIPA) buffer (Sigma) supplemented with 1 mM phenylmethylsulfonyl fluoride (PMSF), 1% protease inhibitor cocktail (Sigma), and 500 U/ml RNase inhibitor (Invitrogen) for 30 min at 4°C. The lysates were clarified by centrifugation at 12,000 g for 15 min at 4°C. Eight µg of anti-ANXA2 antibody (Abcam, ab235939) were conjugated with protein A Magnetic Dynabeads (BioRad, Hercules, CA, United States) under rotary agitation for 1 h. The unbound antibody was then washed out 3 times with PBS-0.1% Tween. The antibody-beads complex was incubated at room temperature with protein samples for 1 h under rotary agitation. The immunoprecipitation (IP) complex was magnetically sorted and washed 3 times with PBS-0.1% Tween according to the manufacturers protocol. The recovered pellet was resuspended: 1) in lysis buffer Qiazol and RNA was extracted with miRNeasy mini kit following manufacturer's protocol (Qiagen), or 2) in Laemmli buffer (Biorad) for protein analysis.

Extracted RNA was retrotranscribed and cDNA was amplified by real time PCR. Specific primers for cel-miR-39 (listed above), hsa-miR-21 (F: TAGCTTATCAGACTGATGTTGA) and hsa-miR-16 (F: GCA​GCA​CGT​AAA​TAT​TGG​CG) were used. qRT-PCR results for the endogenous and exogenous miRNAs were expressed as 2^−ΔCt^, where ΔCt: (Ct treated- Ct SEV) ±SD.

Recovered IP or total SEVs proteins were separated by gel electrophoresis using 4–20% precast gel (Bio-rad) and blotted using the Trans-Blot Turbo Transfer System (Biorad). PVDF membranes were incubated with primary antibody against ANXA2 (ab235939, Abcam), CD63 (Cruz Biotechnology Inc., Santa Cruz, CA, SC5275), CD81 (SC31234), Alix (SC271975) and AGO2 (Abcam, ab32381) as previously described (Iavello et al., 2016). Signal was revealed by incubation with anti-rabbit or anti-goat HRP (Invitrogen) conjugated secondary antibody and ECL substrate (Bio-rad, Hercules, CA, United States). Chemiluminescent signal was acquired on Chemidoc and analyzed with ImageLab (Bio-rad). Experiments were repeated 3 times with similar results.

### *In vitro* Tubulogenesis Assay on HUVEC Cells

HUVEC (15 × 10^4^ cells/well) were seeded onto growth factor reduced Matrigel (BD Biosciences, San Jose, CA, United States)-coated wells and cultured in basal EBM. Cells were treated with 2.5 or 5 × 10^8^ particles/ml (8,000 p/cell or 16,000 p/cell) of SEVs with or without miR-126.

In selected experiments, HUVEC were primed with 10 ng/ml Vascular endothelial growth factors (VEGF) and then treated with 5 × 10^8^ particles/ml of SEVs with miR-19b mimic or with the corresponding dose of miR-19 alone. VEGF-treated (10 ng/ml) HUVEC served as control. Cell organization onto Matrigel was acquired after 24 h by Leica 3,000 microscope (Leica, Wetzlar, Germania). Data were expressed as average of the number of capillaries like structures/field (n = 10 fields) (mean ± SD). Experiments were performed 3 times in duplicates.

### *In vivo* Tubulogenesis and Vessel Recruitment Assay

Animal experiments were performed according to the guidelines for the care and use of research animals and were approved by the local Ethics Committee (authorization number: 490/2015-PR). For tubulogenesis experiments, one million human microvascular endothelial cell (HMEC) pre-stimulated ON (1 × 10^10^ particles co-incubated with 100 pmol mimic) and SEVs (1 × 10^10^ particles) were implanted subcutaneously into severe combined immunodeficient (SCID) mice (Charles River) within Matrigel (n = 3 plugs/condition). After 7 days, mice were sacrificed and Matrigel plugs were recovered. Angiogenesis was calculated as the mean ± SD of the number of vessels with red cells inside per total area of trichromic stained sections (n = 10 fields). For recruitment assay, Friend leukemia virus B (FVB) mice (6–8 weeks old) were subcutaneously injected with growth factor reduced Matrigel (BD Biosciences) mixed with 10 ng/ml VEGF, SEVs (1 × 10^10^ particles), miR-126 (100 pmol), or SEV + miR-126 (1 × 10^10^ particles co-incubated with 100 pmol mimic) (n = 4 plugs/condition). Matrigel alone was used as control (n = 4 plugs). Matrigel plugs were recovered after 7 days and sections were stained by immunofluorescence to detect CD31 expression (anti CD31 antibody, Biomed, Foster City, CA, United States 1:200). Hoechst dye was used to counterstain nuclei. Positive vessels were counted (n = 10 fields), and data were expressed as CD31 positive vessels/field ±SD.

### Statistical Analysis

Statistical analysis was performed using GraphPad V6 software. Results are expressed as mean ± SD unless otherwise reported. Student-t-tests or 1-way ANOVA, followed by Tukey’s or Newman-Keuls multi-comparison tests were applied based on the assay. Statistical significance was set at *p* < 0.05.

## Results

### Characterization of SEVs After Co-incubation With cel-miR-39

**FIGURE 1:**
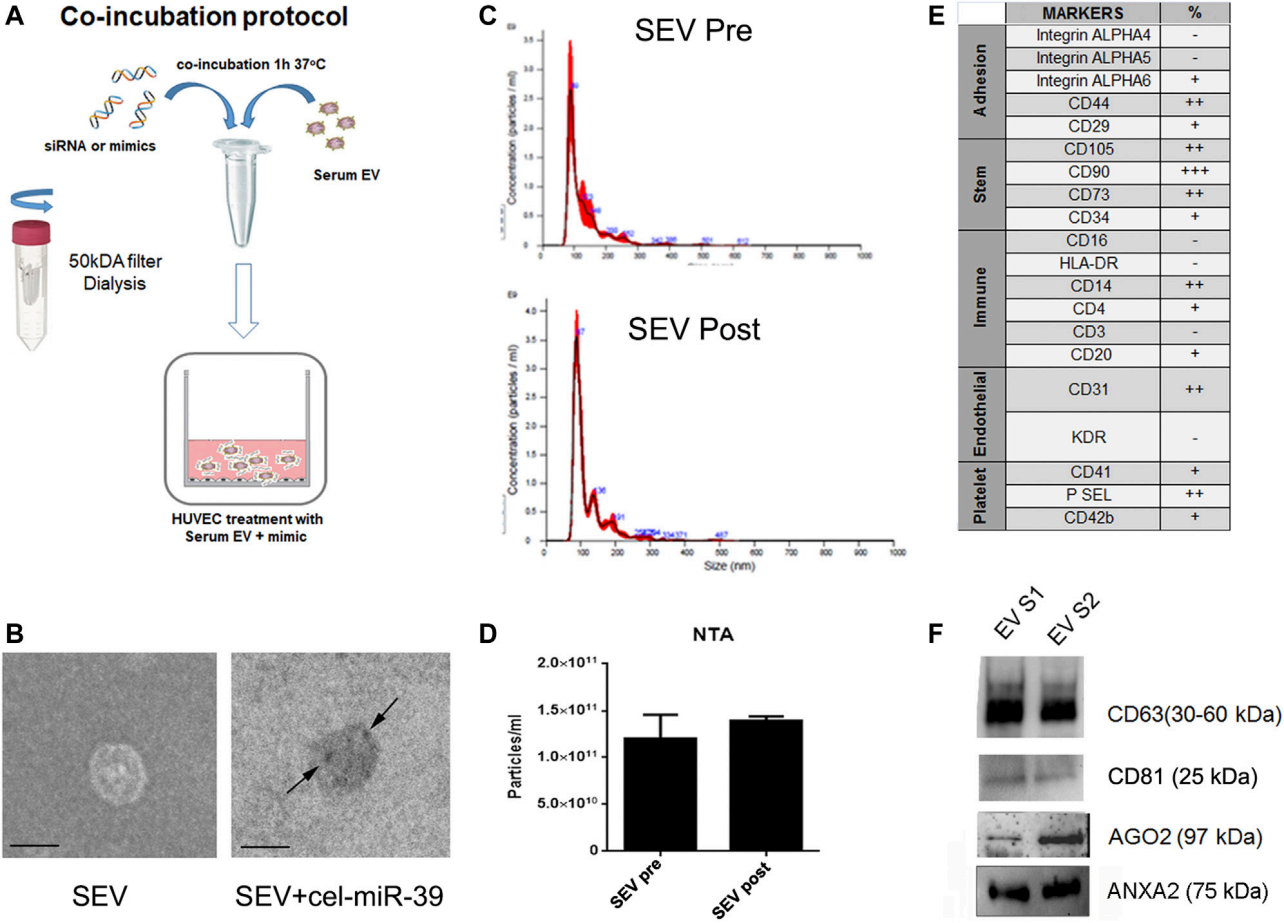
Characterization of SEVs co-incubated with exogenous cel-miR-39. **(A)** Scheme of co-incubation protocol. **(B)** TEM images of SEVs (SEV) and SEV + cel-miR-39. Black arrows indicate the specific bind of cel-miR-39 probe. Black line, 100 nm. **(C)** NTA representative profile of SEVs before (SEV Pre) and after (SEV Post) washing step with 50 kDa concentrator. **(D)** NTA analysis of particles number of SEVs before and after washing step. The same profile and quantification were observed when SEVs were washed with dialysis membrane. Data are expressed as mean ± SD. **(E)** SEV surface marker expression was analyzed by FACS. +: low expression <15%; ++: median expression 15–50%; +++: high expression >50%; - negative expression. **(F)** Western blot analysis of CD63, CD81, Ago2 and ANXA2 on two different SEV pools (EV S1 and EV S2).

The co-incubation protocol used to enrich SEVs with selected mimics is described in Figure 1A. miRNA mimics were co-incubated with SEVs 1 h at 37°C and then washed to eliminate the excess of mimic. The efficiency and specificity of the method were tested using cel-miR-39 mimic, a miRNA from *C. Elegans* not detected in humans. *In-situ* hybridization in combination with TEM were employed to localize the cel-miR-39. As shown in Figure 1B (right panel, black arrow), SEV morphology was maintained after adding the cargo of interest, and cel-miR-39 mimic was detected on the SEV surface. Evaluation by NTA of SEVs before and after the mimic incubation step did not show any significant differences (Figures 1C,D), suggesting that the labeling and washing protocols did not affect SEV dimension and number. The SEV concentration was about 8.75 × 10^11^/ml with a mean size of 184.4 ± 35.5 nm. The expression of the different stem, endothelial, and platelet surface markers was detected in SEVs by flow cytometry, supporting the presence of a heterogeneous population of blood-derived vesicles in our sample (Figure 1E). The expression of CD63 and CD81, exosomal markers, as well as Ago2 and ANXA2, RNA binding proteins (RBPs) was confirmed by western blot analysis (Figure 1F).

### Uptake of Modified SEVs by HUVEC

**FIGURE 2:**
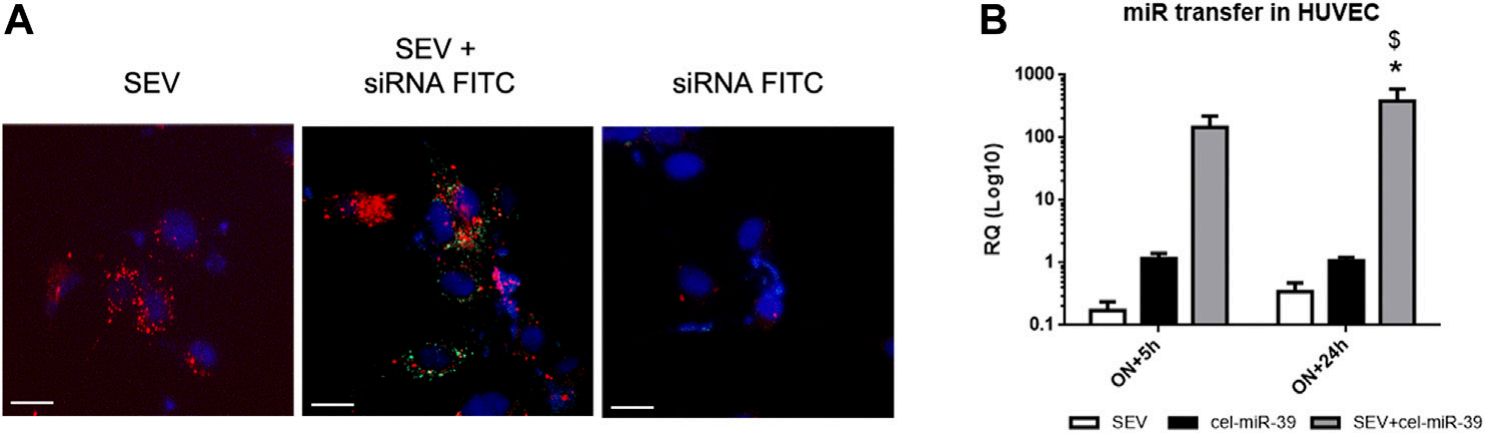
Exogenous mimic delivery by SEVs to HUVEC target cells. **(A)** Incorporation of fluorescent labeled SEVs (red) alone (SEV) and SEVs co-incubated with FITC conjugated control mimic (green) (SEV+ siRNA FITC) or siRNA FITC alone by HUVEC cells. Cell nuclei were stained with Hoechst dye (blue), ×400 magnification. White bar: 10 µm. **(B)** Evaluation of cel-miR-39 transfer into HUVEC by real time PCR. HUVEC were treated overnight (ON) with SEV, cel-miR-39 alone or SEV + cel-miR-39, medium was then replaced, and cells were maintained in culture for other 5 h or 24 h. Data are expressed as RQ ± SD. *p < 0.05 vs. SEV\; $ *p* < 0.05 vs. cel-miR-39.

To monitor the uptake of modified SEVs by target cells, HUVEC were treated with SEVs labeled with Dil lipophilic dye and incubated with FITC conjugates control siRNA (siRNA-FITC). After 24 h both SEVs and SEVs co-incubated with siRNA-FITC (SEV + siRNA FITC) entered HUVEC, whereas siRNA FITC alone did not (Figure 2A). siRNA FITC and Dil SEVs partially co-localized in HUVEC (Figure 2A), suggesting the direct binding of siRNA with SEVs.

To evaluate the transfer of cel-miR-39 mimic into HUVEC, cells were treated with SEVs, SEVs co-incubated with cel-miR-39 (SEV + cel-miR-39) or with mimic alone (cel-miR-39), and miRNA expression was evaluated by real-time PCR. cel-miR-39 expression was detected when HUVEC were treated for ON+5 h with mimic enriched SEVs and remained stable after 24 h after medium replacement. A significantly lower signal of cel-miR-39 was observed when HUVEC were treated with mimic alone (Figure 2B).

### Proangiogenic Effect of Mimic Loaded SEVs *in vitro*

**FIGURE 3:**
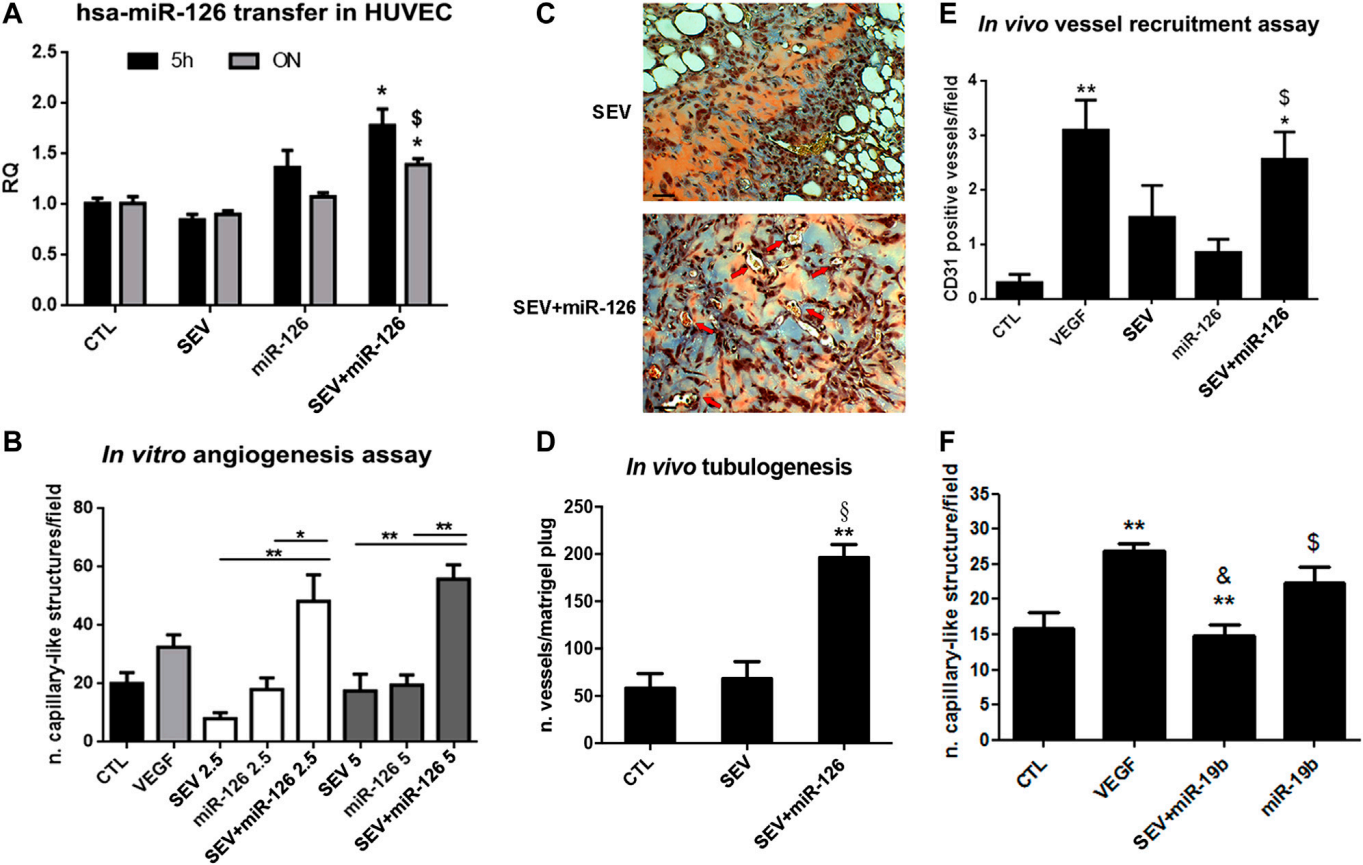
Enhanced angiogenic properties of miR-126 loaded SEVs. **(A)** miR-126 mimic transfer to HUVEC cells. Cells were treated for 5 h or ON with SEVs, miR-126 or SEV + miR-126 and real time PCR was performed. Data are expressed as Relative quantification (RQ)± SD. *p < 0.05 vs. CTL and SEV; $ *p* < 0.05 vs. miR-126. **(B)** *In vitro* capillary-like structure formation assay. HUVEC were treated with different doses of SEVs (2.5 or 5 × 10^8^ p/ml) loaded with miR-126. SEVs and mimic alone were used as internal control. HUVEC treated with VEGF (10 ng/ml) were considered as positive control. Data are expressed as number of capillary-like structure/field (mean ± SD). *p < 0.05 and **p < 0.01 vs. SEV or miR-126. **(C)** Representative micrograph of trichomic staining of HUVEC pre-treated with SEV (left side) or SEV + miR-126 (right side) and injected in Matrigel plugs. The matrigel was recovered after 6 days. Black bar: 50 µm. **(D)** Tubulogenic assay was calculated as average of number of vessel structures/matrigel plug (mean ± SD) *p < 0.05 vs. CTL; §*p* < 0.05 vs. SEV. **(E)** *In vivo* mouse model of vessel recruitment by SEV, SEV + miR-126 and miR-126 embedded in Matrigel plug. Data are expressed as CD31 positive vessels/field (mean ± SD). *p < 0.05 and **p < 0.01 vs. CTL; $ *p* < 0.05 vs. miR-126. **(F)** Antiangiogenic effect of miR-19 loaded SEVs. HUVEC were pre-treated with VEGF (10 ng/ml) and then stimulated with SEVs loaded with miR-19b (SEV + miR-19b) or with miR-19b mimic alone. Data are expressed capillary-like structures/field (mean ± SD). **p < 0.001 vs. CTL; & *p* < 0.01 vs. VEGF; $ *p* < 0.05 vs. EV + miR-19b.

SEVs were incubated with the well-known pro-angiogenic miR-126 (Tiwari et al., 2018). As shown by real-time PCR, miR-126 expression increased when HUVEC were treated for 5 h or ON with SEV + miR-126. On the contrary, any increase was not observed in HUVEC co-incubated with SEVs or miR-126 alone (Figure 3A). By using two different concentrations of SEVs that we demonstrated to be ineffective in the tube formation assay, we observed an induction of capillary-like structure formation only when HUVEC were treated with 2.5 × 10^8^ and 5 × 10^8^ p/ml of SEV + miR-126 (Figure 3B). No effect was observed when HUVEC were stimulated with SEVs or miR-126 alone (Figure 3B). We tested the angiogenic potential of SEV + miR126 also *in vivo*. We demonstrated that endothelial cells pre-treated with SEV + miR-126 have a strong ability to form competent vessels, when inoculated in Matrigel plug *in vivo*, in respect to endothelial cells untreated or treated with SEVs alone (Figures 3C,D). We also tested vessel recruitment capability *in vivo* and we observed that mouse endothelial cells were more prone to invade SEV-miR126 embedded Matrigel plugs to create capillary-like structures in a comparable way of the effect of VEGF embedded Matrigel plugs. On the contrary SEVs or miR-126 alone were unable to induce the same effect (Figure 3E). To test the specificity of the effect mediated by the mimic delivered by SEVs, we used miR-19b that was described to be anti-angiogenic on HUVEC cells (Liang et al., 2018). SEVs co-incubated with miR-19 were able to inhibit the formation of capillary-like structures *in vitro* induced by VEGF, whereas miR-19b alone did not (Figure 3F).

### Evaluation of the Molecules Mediating miRNA Binding to SEVs

**FIGURE 4:**
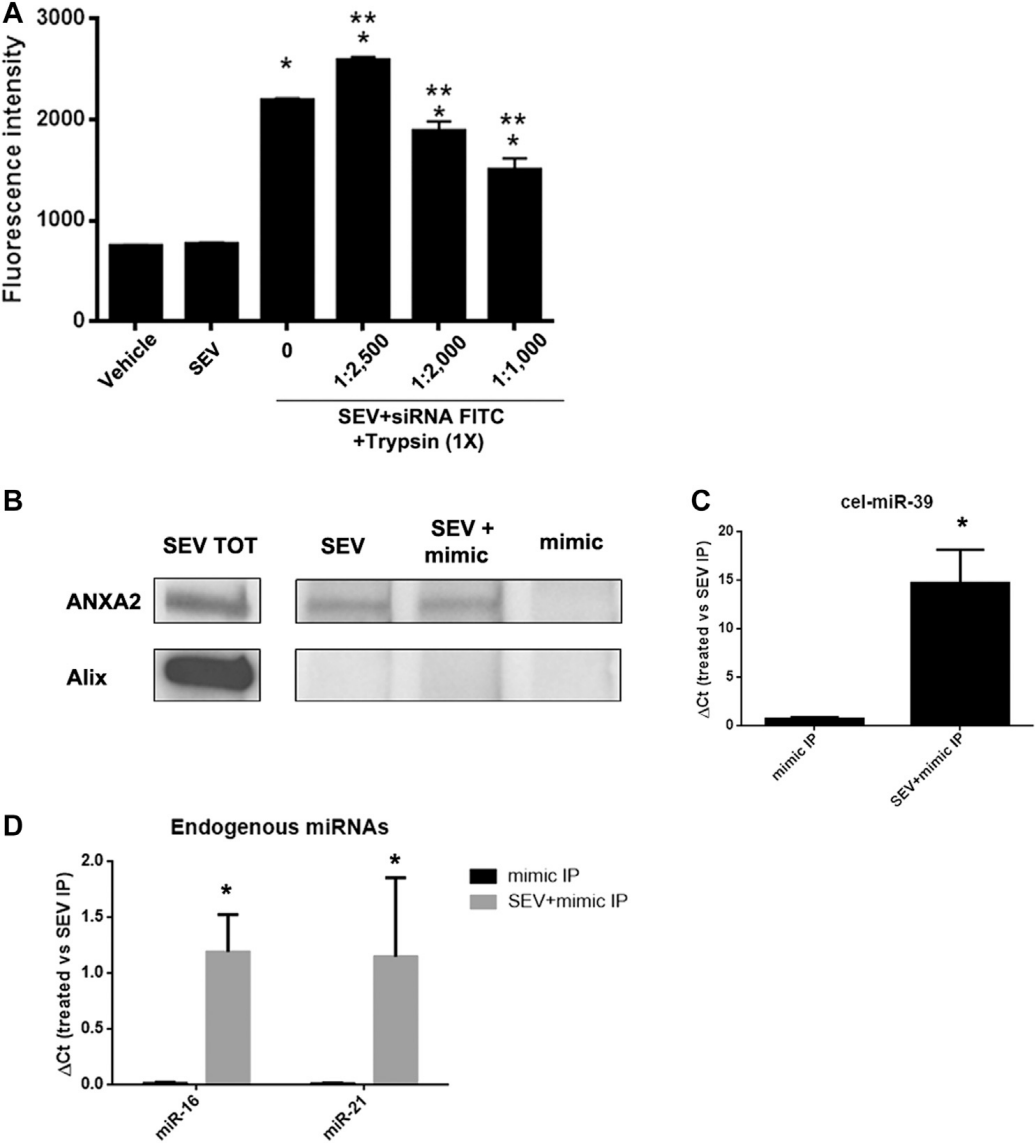
SEVs binding to exogenous mimic is mediated by proteins. **(A)** SEVs loaded with siRNA-FITC were treated with different trypsin concentration (1:1,000 to 1:2,500) for 1 h at 37 C. Fluorimetric analysis were conducted. Data are expressed as fluorescence intensity ±SD the following: *p< 0.05 vs. SEV; **p< 0.05 vs. no trypsin treatment (0). **(B)** ANXA2 western blot analysis of SEVs (SEV), SEV + cel-miR-39 and cel-miR-39 samples, immunoprecipitated for ANXA2. Specificity of the IP procedure was validated by testing ALIX protein presence in the IP. Total protein extract (SEV TOT) was used as positive control for antibody specificity. **(C)** Representative Real time PCR analysis of cel-miR-39 expression detected in SEV + cel-miR-39 and cel-miR-39, immunoprecipitated for ANXA2 (treated: SEV + mimic IP and mimic IP, respectively); *p < 0.05 vs mimic IP. **(D)** Expression of endogenous miR-16 and miR-21 detected in SEV+mimic IP and mimic IP, immunoprecipitated for ANXA2. In all the experiments SEV IP for ANXA2 was used as control. Data are expressed as 2-∆Ct, where ∆Ct = [Ct (treated) - (S EV IP)] ±SD; *p < 0.05 vs. mimic IP.

To evaluate whether the association between miRNAs and SEVs induced by co-incubation was dependent on the interaction with proteins expressed on EV surface, we treated SEVs pre-incubated with FITC-siRNA with different concentrations of trypsin at 37°C for 1 h. We observed a dose-dependent decrease of the fluorescent signal when SEVs were treated with an increased concentration of trypsin (Figure 4A), supporting a protein-dependent binding of exogenous mimic. Since we identified ANXA2 in SEVs (Figure 1F), a protein involved in the delivery of RNAs in a sequence-independent manner (Hagiwara et al., 2015), we investigated its possible role in mediating exogenous delivery of miRNA mimic (cel-miR-39) in our model. IP assay for ANXA2 was performed. Antibody against ANXA2 precipitated the protein of interest as detected by western blot analysis. On the contrary, Alix protein was not precipitated confirming the selectivity of ANXA2 IP (Figure 4B). Moreover, real-time PCR on immunoprecipitated samples showed that cel-miR-39 was strongly detected in SEVs co-incubated with the mimic (Figure 4C), supporting a role of ANXA2 in the binding of cel-miR-39 on SEV surface. We also observed the strong expression of the endogenous miR-21 and miR-16 in SEV + mimic IP and SEVs and their absence in control mimic IP, suggesting that ANXA2 can also bind endogenous miRNAs physiologically transferred by SEVs (Figure 4D).

## Discussion

EVs have been identified as new players in intercellular communication able to deliver a different type of active molecules to target cells. The use of EVs from different sources, as vehicles to deliver exogenous therapeutic molecules, is very challenging (Bei et al., 2017; Pang et al., 2020). In the present study, we loaded exogenous miRNA mimics on SEVs by a temperature-controlled co-incubation procedure followed by dialysis or filtration purification. The efficacy of the procedure was demonstrated by the detection of cel-miR-39 mimic on EV surface by electron microscopy, by mimic-conjugated EV uptake analysis by fluorescence microscopy and real-time PCR. Moreover, we demonstrated, that the transfer of an angiogenic miRNA to HUVEC cells through SEVs was able to induce a stable pro-angiogenic response both *in vitro* and *in vivo*.

In the last decades, many methods were developed to load exogenous molecules into EVs, but most of the systems although demonstrating loading capacity, were therapeutically inefficient (Kooijmans et al., 2013; Momen-Heravi et al., 2014). For example, the increase in membrane permeability triggered by electroporation may induce loss of critical active molecules in EVs with a native regenerative activity such as those derived from stem cells (Kooijmans et al., 2013). For instance, EVs were also described in different body fluids such as serum, plasma, milk, and saliva (Zaborowski et al., 2015). These biological matrixes can be useful candidates to isolate a high amount of EVs for delivery purposes. In our experimental protocol, EVs were isolated from the serum of healthy subjects. They were heterogeneous in size, as detected by NTA analysis. Indeed, they carried several surface molecules, associated with platelets, endothelial, and immune cells derivation and expressed tetraspanin markers. Moreover, the RBPs, Ago2 and ANXA2 proteins were also present, demonstrating a diverse phenotype characteristic of both microvesicles and exosomes in our preparation. Electroporation of plasma EVs was developed by appropriate settings by Pomatto et al. (Pomatto et al., 2019), to enrich them with exogenous antitumor miRNAs. Despite the successful procedure, a miRNA loading on the EV surface can be an easy vehiculation mechanism without any physical modification to the EV structure and membranes. In our experiments, SEVs loaded with exogenous mimics were able to maintain the same number and dimension after the coincubation and filtration procedures. Mimic surface localization was confirmed by *in situ* hybridization detected by transmission electron microscopy. Moreover, SEVs co-incubated with an exogenous fluorescent or synthetic mimic, were taken up by HUVEC cells and the mimic localized in the cell cytoplasm being steady until 24 h post-treatment.

EVs derived from different stem cells were shown to display angiogenic properties (Deregibus et al., 2007; Bian et al., 2014). For instance, hypoxia primed mesenchymal stem cells (hyMSCs) derived EVs were described to protect cardiac and renal tissues from ischemic injury, promoting blood vessel formation and preserving cardiac and renal performance (Zhu et al., 2018; Collino et al., 2019). *In vitro* hyMSC EVs enhanced proliferation, migration, and tube formation of endothelial cells (Zhu et al., 2018; Collino et al., 2019). Interestingly, endothelial progenitor cell-derived EVs were also able to stimulate neovascularization, endothelial repair, and regeneration in a peripheral artery disease mouse model (Ranghino et al., 2012). The pro-angiogenic properties of SEVs were also demonstrated in the same model (Cavallari et al., 2017). SEVs from healthy donors displayed an angiogenic potential, enhancing capillary formation via TGFβ1 signaling, both *in vitro* and *in vivo* (Cavallari et al., 2017).

miRNAs can also participate in the angiogenesis process. Different miRNAs such as miR-126, miR-221/222, miR-17-92 cluster, miR-93 can regulate endothelial response to angiogenic stimuli interacting with different cell signaling pathways (Tiwari et al., 2018). Among angiomiRs, we selected miR-126 that controls vascular integrity and angiogenesis by promoting MAP kinase and PI3K signaling in response to VEGF and FGF (Wang and Olson, 2009). miR-126 loaded SEVs was uptaken by endothelial cells as shown by transfer experiments and enhanced their pro-angiogenic potential by inducing capillary-like structure formation with respect to SEVs alone when applied in a concentration below their effective dose (Cavallari et al., 2017). Moreover, when miR-126 loaded SEVs were mixed with Matrigel plugs they were able to stimulate the recruitment of mouse endothelial cells *in vivo* with respect to naïve SEVs promoting neo-angiogenesis.

The stable environment provided by SEVs in the delivery of RNA mimic is an interesting issue to be addressed. Lately, many RNA interacting proteins (RIPs) have been described to be present in different EV sources (Jeppesen et al., 2019). Frequently described RIPs are Ago1–4, ANXA2, RPS3, RPS8, EEF2, EEF1A1, MVP, PARK7/DJ1, hnRNPA2B1, and GAPDH (Hagiwara et al., 2015; Mateescu et al., 2017). We here hypothesize that the delivery of the exogenous mimic can be controlled by proteins on the EV surface, as their anchorage was susceptible to trypsin proteolysis. Among RIPs, ANXA2 was described as a protein involved in the regulation of the delivery of mimic inside EVs without a sequence motif recognition (Hagiwara et al., 2015). We demonstrated that SEVs expressed high levels of ANXA2. Moreover, cel-miR-39 mimic was able to co-immunoprecipitate with ANXA2 in miRNA loaded SEVs, suggesting a role of ANXA2 in the delivery of exogenous miRNAs as detected by real-time PCR analysis. Interestingly endogenous miR-21 and miR-16 also co-immunoprecipitated with ANXA2, both in naïve and cel-miR-39 loaded EVs, supporting a physiological role of ANXA2 in RNA package in EVs. It can be speculated that other proteins have the same adhesive properties, not providing a selective role to ANXA2 in this process. One limitation of the proposed methodology is the possibility that loaded mimic can be susceptible to physiological RNAse activity. However, the presence of a mimic-protein interaction can partially overcome the degradation process. Further studies need to be performed to translate the method to clinical practice.

In conclusion, our results suggest that coincubation is a promising alternative method for the EV cargo enrichment maintaining EV integrity and stability. Moreover, by the delivery of miR-126, we were able to enhance the pro-angiogenic potential of SEVs both *in vitro* and *in vivo*, showing that this system is sufficiently stable to enter target cells and exploit a function. Moreover, the RBPs on EV surface can act as a natural platform to enhance the EV intrinsic therapeutic potential or to provide an alternative function to ineffective EVs.

## Data Availability

The raw data supporting the conclusions of this article will be made available by the authors, without undue reservation.

## Abbreviations

ANXA2, Annexin A2; APC, Allophycocyanin; DMSO, Dimethyl sulfoxide; EBM, Endothelial Basal Medium; FITC, Fluorescein isothiocyanate; FVB, Friend leukemia virus B mice strain; HMEC, Human Microvascular Endothelial Cells; HUVEC, Human Umbilical Vein Endothelial Cells; IP, Immunoprecipitation; miRNA, microRNA; NTA, Nanosight Analysis; PBS, Phosphate Buffer Saline; PE, Phycoerythrin; RIPA, Radio immunoprecipitation assay buffer; RBPs/RIPs, RNA binding proteins/RNA interacting proteins; RPMI, Roswell Park Memorial Institute; SCID, Severe Combined Immunodeficient mice; SEVs, Serum derived Extracellular Vesicles; siRNA, small interfiring RNA; TEM, Transmission Electron Microscopy; VEGF, Vascular endothelial growth factors.
